# Chronotype, Sleep, and Depressive Symptoms Among Chinese College Students: A Cross-Sectional Study

**DOI:** 10.3389/fneur.2020.592825

**Published:** 2020-12-17

**Authors:** Tingting Li, Yang Xie, Shuman Tao, Yajuan Yang, Honglv Xu, Liwei Zou, Fangbiao Tao, Xiaoyan Wu

**Affiliations:** ^1^Department of Maternal, Child and Adolescent Health, School of Public Health, Anhui Medical University, Hefei, China; ^2^MOE Key Laboratory of Population Health Across Life Cycle, Hefei, China; ^3^NHC Key Laboratory of Study on Abnormal Gametes and Reproductive Tract, Hefei, China; ^4^Anhui Provincial Key Laboratory of Population Health and Aristogenics, Anhui Medical University, Hefei, China; ^5^Department of Nephrology, The Second Hospital of Anhui Medical University, Hefei, China; ^6^School of Nursing, Anhui Medical University, Hefei, China

**Keywords:** chronotype, depressive symptoms, sleep quality, circadian rhythms, college students

## Abstract

**Objective:** To describe the prevalence of chronotype and depressive symptoms among Chinese college students and to examine the association between chronotype and depressive symptoms.

**Methods:** From April to May 2019, a cross-sectional survey was conducted among 1,179 Chinese college students from 2 universities in Anhui and Jiangxi provinces. A total of 1,135 valid questionnaires were collected, the valid response rate was 98.6%. The questionnaire investigated age, gender, major, height, weight, only child status, living place, self-reported family economy, and self-reported study burden. The chronotype was assessed by the Morning and Evening Questionnaire (MEQ). Depressive symptoms and sleep quality were evaluated by the Patient Health Questionnaire 9 (PHQ-9) and the Pittsburgh Sleep Quality Index (PSQI), respectively. A Chi-square test was used to examine the proportion of depressive symptoms among Chinese college students with different demographic characteristics. The generalized linear model was used to analyze the relationships between chronotype and depressive symptoms.

**Results:** The proportion of morning types (M-types), neutral types (N-types), and evening types (E-types) of college students were 18.4, 71.1, and 10.5%, respectively. The proportion of mild depression, moderate depression, and moderate to severe depression of participants were 32.4, 6.0, and 4.2%, respectively. Compared to the M-types, after controlled for age, gender, major, sleep quality, self-reported study burden, father's education level, and self-reported family economy, depressive symptoms were positively correlated with E-types (*OR* = 2.36, 95% CI: 1.49–3.73).

**Conclusions:** There was a significant association between chronotype and depressive symptoms among Chinese college students. Further longitudinal studies were needed to clarify the causal relationship between chronotype and depressive symptoms.

## Introduction

Chronotype was a unique personal biological clock system that was determined by daytime activities and bedtime preferences. The cyclic factor that determines this preference was called a circadian preference, which largely depends on an individual's endogenous component (1). Circadian preference was a continuum but was usually divided into three chronotypes: morning types (M-types), neutral types (N-types), and evening types (E-types) (2). At the onset of adolescence, a sharp shift toward E-types starts, reaching its peak at the end of youth, followed by a steady shift toward M-types as the aging process occurs (3, 4). In a large student sample, the proportion of E-types was 24% and was higher than M-types (16%) (5).

Chronotype affects the psychological health of individuals. Studies have shown that E-types have been associated with increased risk for depressive symptoms (6), and people who stay up late were acknowledged to be more likely to experience depressive symptoms (7). Other studies have shown that E-types have been related to many adverse health outcomes, including mental and physical health problems (8, 9). Besides, E-types can be highly impulsive and use more fatal suicide methods than M-types (10). Thus, the inclination to be an M-types was generally recognized as a protective factor. In contrast, the propensity to be an E-types was a risk factor for triggering personality features associated with a mental disorder (1).

The risk of depressive symptoms sharply rises as a transition from childhood to adolescence. Meanwhile, college students undergo significant changes during campus life due to free of parent-imposed constraints in China. Thus, their lifestyle behaviors can be unhealthy, such as late sleeping, extended screen time, and lack of physical activity (11). In addition to lifestyle changes, many college students also deal with novel challenges arising from adolescent physiological changes, such as a biologically driven delayed sleep phase, which may lead to adverse health outcomes (12). There was evidence suggests that college students were at high risk of depressive symptoms, despite being socially advantaged. Studies have shown that the overall prevalence of depressive symptoms among college students was 52.6% (13). Furthermore, epidemiology studies have indicated that college students with higher levels of depressive symptoms tend to encounter an increased risk of adverse events such as poor academic performance (14), higher levels of substance use (15), and higher levels of suicide (16).

Previous studies have demonstrated that adolescents with E-types have an increased risk of depressive symptoms (17, 18). Two studies conducted among college students indicated that E-types were more likely than M-types to report depressive symptoms (19, 20). However, studies conducted in patients found inconsistent results, which failed to find an association between E-types and depressive symptoms (21, 22). Given the higher prevalence of late bedtimes and the higher risk of depressive symptoms among college students, we conducted an epidemiological investigation of the association between chronotype and depressive symptoms among Chinese college students to provide evidence for further prevention and control of depression in college students.

## Methods

### Participants

A total of 1,179 college students were recruited from a medical university and a comprehensive normal university located in Hefei, Anhui Province, and Shangrao, Jiangxi Province, using stratified cluster sampling between April to May 2019. Firstly, two cities were selected by convenient sampling. Then, two universities were based on stratified cluster sampling. Lastly, faculties and classes were selected randomly from the selected universities. Teachers and professional investigators distributed a quick response code to the students for scanning by using their cell phones to complete the electronic questionnaires. A total of 1,135 valid respondents were analyzed, and the response rate was 98.6%.

The current study was approved by the Ethics Committee of Anhui Medical University. Written informed consent was obtained from all of the participants.

### Sociodemographic Data

A self-administered questionnaire, including information on sociodemographic indicators, height, weight, chronotype, depressive symptoms, and sleep quality was administered during a 10–20 min session in the classroom. The following sociodemographic characteristics were obtained: age, gender, only child status, living place (urban, rural area), self-reported family economy (low, high), and self-reported study burden (low, high).

### Chronotype

Chronotype was assessed by the Morning and Evening Questionnaire (MEQ). The MEQ was the validity and high-reliability tool used to describe chronotype of sleep or phase preferences and was the most widely used tool for identifying chronotype (23). This study used MEQ-5 to assessed the chronotype of college students. The total score ranges from 4 to 25 points. According to the score, chronotype can be divided into three types: E-types (4–11 points), N-types (12–17 points), and M-types (18–25 points) (24). Cronbach's α in this study was 0.68.

### Depressive Symptoms

Depressive symptoms were evaluated by the Patient Health Questionnaire 9 (PHQ-9). The PHQ-9 scale contains nine items, which cover the experience of pleasure, feeling down, sleep disruption, energy levels, appetite, feeling a failure, trouble concentrating, speaking slowly or being fidgety, and having negative thoughts around suicide or self-harm over the previous 2 weeks (25). The total score ranges from 0 to 27 points. According to the score, depressive symptoms can be divided into four types: no depression (4–11 points), mild depression (5–9 points), moderate depression (10–14 points), moderate to severe depression (15–27 points) (26). Cronbach's α in this study was 0.81.

### Sleep Quality

Sleep quality was evaluated by the Pittsburgh Sleep Quality Index (PSQI). Nineteen individual items generate seven component scores: subjective quality of sleep, sleep latency, sleep duration, sleep efficiency, sleep disorders, medication use, and daytime dysfunction (27). The sum of these seven components' scores yields one global score, the PSQI scores, ranging from 0 to 21. According to the score, sleep quality can be divided into two types: sleep quality good (0–7 points) and sleep quality poor (8–21 points) (27). Cronbach's α in this study was 0.71.

### Statistical Analysis

Statistical analysis was performed using SPSS version 23.0 (Statistical Package for the Social Sciences). The Chi-square test was performed to compare the incidence of depressive symptoms among different sociodemographic variables, chronotype, and sleep quality. The generalized linear model was used to analyze the relationships between chronotype and depressive symptoms. Odds ratios (*OR*s) and 95% confidence intervals (95% *CI*s) were calculated for the explanatory factors and adjusted for confounding factors, including age, gender, major, sleep quality, self-reported study burden, father's education level, and self-reported family economy. Statistical significance was set at *P* < 0.05.

## Results

### Characteristics of Participants

Table 1 displays the characteristics and group differences of 1,135 college students aged between 15 and 26 years old (mean ± SD: 18.8 : 1.2 years), 432 were males (38.1%), and 703 were females (61.9%).

**Table 1:** Characteristics of depressive symptoms in college students (%).

**Variable**	**n**	**PHQ-9**				**χ^2^-value**
		**No depression**	**Mild depression**	**Moderate depression**	**Moderate to severe depression**	
Gender						1.96
Male	432	245 (56.7)	136 (31.5)	30 (6.9)	21 (4.9)	
Female	703	406 (57.8)	232 (33.0)	38 (5.4)	27 (3.8)	
Major						26.10^b^
School of public health	232	150 (64.7)	66 (28.4)	12 (5.2)	4 (1.7)	
School of nursing	334	201 (60.2)	112 (33.5)	14 (4.2)	7 (2.1)	
School of chemistry and environmental sciences	265	141 (53.2)	92 (34.8)	16 (6.0)	16 (6.0)	
School of sports	304	159 (52.3)	98 (32.2)	26 (8.6)	21 (6.9)	
Self-reported study burden						16.40^a^
Low	21	11 (52.4)	8 (38.1)	0 (0.0)	2 (9.5)	
Medium	695	420 (60.4)	209 (30.1)	46 (6.6)	20 (2.9)	
High	419	220 (52.5)	151 (36.0)	22 (5.3)	26 (6.2)	
Living place						5.04
Rural	633	347 (54.8)	222 (35.1)	39 (6.2)	25 (3.9)	
Urban	502	304 (60.5)	146 (29.1)	29 (5.8)	23 (4.6)	
Only child status						0.49
Yes	268	158 (59.0)	84 (31.3)	16 (6.0)	10 (3.7)	
No	867	493 (56.9)	284 (32.8)	52 (6.0)	38 (4.3)	
Father's education level						19.70^b^
Primary school and below	257	130 (50.6)	88 (34.2)	18 (7.0)	21 (8.2)	
Middle school	539	306 (56.8)	182 (33.8)	32 (5.9)	19 (3.5)	
Senior high school and above	339	215 (63.4)	98 (28.9)	18 (5.3)	8 (2.4)	
Mother's education level						7.09
Primary school and below	497	270 (54.3)	171 (34.4)	31 (6.3)	25 (5.0)	
Middle school	396	227 (57.3)	132 (33.3)	22 (5.6)	15 (3.8)	
Senior high school and above	242	154 (63.6)	65 (26.9)	15 (6.2)	8 (3.3)	
Self-reported family economy						14.08^a^
Low	272	139 (51.1)	98 (36.0)	15 (5.5)	20 (7.4)	
Medium	800	470 (58.8)	255 (31.9)	49 (6.1)	26 (3.2)	
High	63	42 (66.7)	15 (23.8)	4 (6.3)	2 (3.2)	

a *P-value < 0.05*.

b *P-value < 0.001*.

Of the 1,135 participants, the proportion of mild depression, moderate depression, moderate to severe depression of college students were 32.4, 6.0, and 4.2%, respectively. However, there were no sex-based differences in depressive symptoms (*P* = 0.581). Depressive symptoms revealed no statistically significant differences by living place (*P* = 0.441), only child status (*P* = 0.921), and mother's education level (*P* = 0.312). College students were from study burden high, a family with a low self-reported family economic status or father's education level low showed higher rates of depressive symptoms. The difference was statistically significant (*P* < 0.05). Moreover, compared to other majors, the school of sports' college students showed higher rates of depressive symptoms (*P* = 0.002).

### The Distribution Characteristics of Chronotype and Depressive Symptoms

The proportion of M-types, N-types, and E-types were 18.4, 71.1, and 10.5%, respectively. The proportion of depressive symptoms in E-types was 56.3% and was higher than M-types (34.4%) and N-types (42.8%). Compared to the M-types and N-types, there were fewer cases of no depression and mild depression in the E-types (Table 2). Compared to the M-types, there were more cases of moderate depression and moderate to severe depression in the E-types (Table 2). The difference was statistically significant (*P* < 0.05).

**Table 2:** Distribution characteristics of chronotype and depressive symptoms.

**Depressive symptoms**	**n**	**Chronotype**			**χ^2^-value**
		**M-types**	**N-types**	**E-types**	
PHQ-9					19.47^a^
No depression	651	137 (21.0)	462 (71.0)	52 (8.0)	
Mild depression	368	54 (14.7)	268 (72.8)	46 (12.5)	
Moderate depression	68	9 (13.2)	48 (70.6)	11 (16.2)	
Moderate to severe depression	48	9 (18.8)	29 (60.4)	10 (20.8)	

a *P-value < 0.05*.

### Associations of Chronotype, Sleep Quality, and Depressive Symptoms

The proportion of poor sleep quality in M-types, N-types, and E-types were 9.6, 13.3, and 20.2%, respectively. In the M-types and E-types, college students with moderate depression and moderate to severe depression were more likely to have poor sleep quality than those with no depression and mild depression (Table 3). In the N-types, college students with mild depression, moderate depression, and moderate to severe depression were more likely to have poor sleep quality than those with no depression (Table 3). The difference was statistically significant (*P* < 0.05).

**Table 3:** Associations of chronotype, sleep quality and depressive symptoms in college students.

**Chronotype**	**Sleep quality**	**n**	**PHQ-9**				**χ^2^*-*value**
			**No depression**	**Mild depression**	**Moderate depression**	**Moderate to severe depression**	
M-types	Good	189	131 (69.3)	49 (25.9)	7 (3.7)	2 (1.1)	54.32^b^
	Poor	20	6 (30.0)	5 (25.0)	2 (10.0)	7 (35.0)	
N-types	Good	700	444 (63.4)	217 (31.0)	29 (4.1)	10 (1.4)	140.76^b^
	Poor	107	18 (16.8)	51 (47.7)	19 (17.8)	19 (17.8)	
E-types	Good	95	49 (51.6)	39 (41.1)	4 (4.2)	3 (3.2)	35.73^b^
	Poor	24	3 (12.5)	7 (29.2)	7 (29.2)	7 (29.2)	

b *P-value < 0.001*.

### Generalized Linear Model Analysis of Chronotype and Depressive Symptoms

The generalized linear model analysis indicated that depressive symptoms of college students were statistically positively correlated with N-types (*OR* = 1.38, 95%CI: 1.01–1.88) and E-types (*OR* = 2.48, 95%CI: 1.60–3.85) (Table 4). After controlled for age, gender, major, sleep quality, self-reported study burden, father's education level, and self-reported family economy, depressive symptoms of college students were positively correlated with E-types (*OR* = 2.36, 95%CI: 1.49–3.73) (Table 4). The association was statistically significant (*P* < 0.05).

**Table 4:** Generalized linear model analysis of chronotype and depressive symptoms.

**Chronotype**	**PHQ-9**	
	**Crude *OR* (95% *CI*)**	**Adjusted *OR* (95% *CI*)**
M-types	1.00	1.00
N-types	1.38 (1.01–1.88)^a^	1.33 (0.96–1.84)
E-types	2.48 (1.60–3.85)^b^	2.36 (1.49–3.73)^a^

*Adjusted for age, gender, major, sleep quality, self-reported study burden, father's education level, and self-reported family economy*.

a *P < 0.05*.

b *P < 0.001 compared with the referent*.

## Discussion

To our knowledge, this was the first study conducted to examine the association between chronotype and depressive symptoms among Chinese college students. We found that E-types were positively correlated with depressive symptoms of college students. Furthermore, we also found that sleep problems play a significant role in the association between E-types and depressive symptoms. Compared to the general population, there appeared a high frequency of individuals at risk of depressive symptoms in the sample (42.6%), including mild depression (32.4%), moderate depression (6.0%), and moderate to severe depression (4.2%). However, the mean prevalence of depressive symptoms of college students was 30.6%, based on previous studies (28). Furthermore, in the present study, the proportion of depressive symptoms in E-types was 56.3% and was higher than M-types (34.4%) and N-types (42.8%). However, other research found most depressed individuals to be N-types (29).

There was a rapid transformation toward E-types in modern society due to increased technological preferences, with a substantial effect on chronotype (30). Meanwhile, with increased age and adolescence development, college students showed significant eveningness chronotype due to adolescent physiological changes such as a biologically driven delayed sleep phase (31). In the present study, the proportion of M-types (18.4%) was higher than E-types (10.5%). However, in another study of college students, the proportion of E-types (28.6%) was higher than M-types (12.7%) (32). Furthermore, in the present study, the proportion of poor sleep quality in E-types was 20.2% and was higher than M-types (9.6%) and N-types (13.3%). The current results were consistent with previously reported associations between chronotype and sleep quality (33).

Adolescence and young adulthood were associated with an E-types orientation, which could be due to social factors and developmental maturation processes (34). Also, circadian rhythm might be affected by college students' lifestyle, potential addictions, and general habits. Nowadays, an emerging body of evidence has shown the impact of caffeinated beverages in disrupting an individual's preferred sleep timing or chronotype (35–37). However, college students who were extreme E-types may voluntarily shorten their hours of sleep in response to exams, review lessons, or engage in entertainment and social contact purposes. Notably, additional caffeinated intake was acquired to maintain focus. Previous studies have also found that light exposure was considered an essential zeitgeber in circadian systems (38), affecting melatonin secretion and extending the entrainment phase, thereby developing E-types (39). As for college students, they will spend more time staying indoors (such as classrooms and dorms) than outdoors and generally experience a zeitgeber reduction because they were exposed to less light during the daytime. Furthermore, studies had also shown that using a mobile phone for playing, surfing, and texting in bed before sleep was associated with a relative eveningness chronotype (40).

At the same time, the risk of depressive symptoms of college students increases sharply (41). Previous studies have also shown that circadian rhythm and sleep disruptions may have a significant role in the vulnerability to mood disorders and the precipitation of disorder symptoms (42). Yet little research has examined the effect these changes can have on college students' mental health and the role that chronotype plays in this process. The current study revealed E-types were positively correlated with depressive symptoms of college students. Similarly, a Croatia study has shown that E-types have been associated with depressive symptoms of college students (43). In a Dutch college student study, E-types can predict more depressive symptoms (β = −0.082, *P* = 0.028) (44). Furthermore, E-types individuals were more likely to report a past diagnosis of a depressive disorder and an earlier onset of depressive symptoms among college students (45). Hence, E-types appears to be an independent risk factor for depressive symptoms among college students, though more studies were warranted to confirm this observation.

Moreover, studies have shown that lifestyle-related risk factors can contribute to depressive symptoms, such as screen time, unhealthy diets, sedentary lifestyles, stressful events, physical activity, and sleep problems (46). Sleep problems have been proposed to play a mediating role in the association between E-types and depressive symptoms (47). Studies have shown that E-types were associated with shorter sleep duration, poorer sleep quality, and insufficient sleep (48). Compared to the M-types and N-types, there were more poor sleep quality cases in the E-types in the present study. College students with moderate depression and moderate to severe depression were more likely to have poor sleep quality than those with no depression. In general, E-types were more likely to suffer from sleep problems. Depressive symptoms and sleep problems tend to interact with each other in many cases. Thus, sleep problems can play a significant role in the depressive symptoms experienced by E-types.

People who were E-types were more likely to have depressive symptoms. The main mechanism underlying chronotype and mood problems seems to involve variations in biological clock genes (*CLOCK, PER1*, and *PER2*) (43). Biological clock genes play an essential role in the critical period of adolescent brain development. Their abnormal expression may change the temporal structure of teenage brain maturation and development, which may lead to dysrhythmia and abnormality of biological rhythm, thus weakening the synchronization between internal and external rhythms, leading to the occurrence of depressive symptoms (49). Furthermore, the underlying mechanisms linking E-types and depressive symptoms have also been explored. E-types have been associated with a lower behavioral activation system, which in turn leads to lower reward responsiveness and lower positive affect, and consequently depressive symptoms (50).

The strengths of the present study include the large sample that has been included in the study, which may make our findings convincing. In addition, we used the generalized linear model to better estimate the associations between chronotype and depressive symptoms. Despite the above strengths, our study has several limitations. First, the cross-sectional survey limits the power with which the causal relationships can be determined. Further longitudinal studies were needed to clarify the causal relationships of chronotype and depressive symptoms. Second, self-reported questionnaires might not allow drawing solid consequences. Third, self-reported depressive symptoms may differ from clinically diagnosed criteria for depressive symptoms. Finally, the relationship between chronotype and depressive symptoms were known findings internationally. However, this was the first time explored by Chinese college students.

## Conclusion

This study showed a significant correlation between eveningness chronotype and depressive symptoms among Chinese college students. Moreover, college students with depressive symptoms were more likely to have poor sleep quality than those without. Therefore, depressive symptoms prevention efforts that examine both eveningness chronotype and sleep quality were vital for early detection of depression among college students.

## Data Availability Statement

The original contributions presented in the study are included in the article/supplementary materials, further inquiries can be directed to the corresponding author/s.

## Ethics Statement

The studies involving human participants were reviewed and approved by the Ethics Committee of Anhui Medical University. The patients/participants provided their written informed consent to participate in this study.

## Author Contributions

XW and FT conceived and designed the experiments. ST and YY performed the experiments. TL, YX, and LZ analyzed the data. HX contributed reagents, materials, and analysis tools. TL wrote the paper. TL contributed to study design. All authors who contributed to the manuscript gave their approval for its submission to Frontier in neurology. The work presented here has not been published previously and is not being considered for publication elsewhere. The author(s) read and approved the final manuscript.

## Conflict of Interest

The authors declare that the research was conducted in the absence of any commercial or financial relationships that could be construed as a potential conflict of interest.
